# Black American Fathers Employed in Higher-Risk Contexts for Contracting COVID-19: Implications for Individual Wellbeing and Work-Family Spillover

**DOI:** 10.1177/15579883211005617

**Published:** 2021-04-13

**Authors:** Shauna M. Cooper, Alvin Thomas, Olajide Bamishigbin

**Affiliations:** 1 University of North Carolina at Chapel Hill, Chapel Hill, NC, USA; 2 University of Wisconsin-Madison, Madison, WI, USA; 3 California State University, Long Beach, CA, USA

**Keywords:** family functioning, psychosocial and cultural issues, fathering, parenting, social determinants of health, behavioral research, research, men’s health

## Abstract

Black Americans remain disproportionately affected by the COVID-19 pandemic. Emerging data suggests that employment in certain occupations (e.g., essential; frontline) may place individuals at higher-risk for contracting COVID-19. The current investigation examined how Black American fathers’ COVID-19 perceived work risk was associated with their individual well-being (COVID-19 diagnosis; depressive and anxiety symptoms; sleep disturbance; sleep quality) as well as spillover into family contexts. Participants were 466 Black American fathers (*M* = 36.63; *SD* = 11.00) who completed online surveys in June–July 2020. Adjusted binomial logistic and multiple regressions were estimated to examine how fathers’ work context was associated with COVID-19 health outcomes, psychological functioning, sleep health, and family stress. Descriptive analyses revealed that 32% of fathers reported a personal diagnosis of COVID-19 and 21% indicated that an immediate family member had been diagnosed. Adjusted binomial logistic regression analyses revealed that fathers working in higher-risk contexts for contracting COVID-19 had a greater odds ratio for both a personal (OR: 1.68, 95% CI: 1.05, 2.68) and an immediate family member diagnosis (OR: 2.58, 95% CI: 1.52, 4.36). Working in a higher-risk context for contracting COVID-19 was associated with poorer psychological functioning, greater sleep disturbance, and higher levels of family discord. Findings suggest that Black fathers working in higher risk contexts may be at risk for COVID-19 exposure and infection. Further, this study indicates that these effects extend to their own well-being, including mental and sleep health as well as increased family stress.

Over 30 million people have been diagnosed with COVID-19 and over 500,000 deaths have been attributed to COVID-19 related complications in the United States (Center for Disease Control, 2021). Research has revealed that, compared to White and Asian Americans, Black Americans have higher COVID-19 mortality rates (APM Research Lab, 2021; Sneed et al., 2020). Current statistical data indicates that 1 out of 555 Black Americans has died from COVID-19 (APM Research Lab, 2021). Although within group data highlighting gender differences remain sparse, U.S. men, across all racial groups, are more likely to suffer negative health consequences associated with COVID-19 in comparison to women (Center for Disease Control, 2021; Mutambudzi, 2020; Treadwell, 2020). International studies have revealed that Black men, after adjusting for key demographic variables, are almost two times as likely to die after contracting COVID-19 than White men (Ayoubkhani et al., 2020). Among the general population, a number of risk factors have been identified, including preexisting health conditions and older age (Garg, 2020). Occupations and work contexts also have been associated with increased risk of being exposed to and contracting COVID-19 (Lancet, 2020; Larochelle, 2020; Rho et al., 2020; Thompson, 2020). Studies report that essential workers are at greater risk for COVID-19 when compared to nonessential workers (Milligan et al., 2020; Mutambudzi, 2020; Roberts, 2020; Rogers, 2020). Though robust transmission rate data are still emerging, state- and county-level data indicate that essential and frontline workers as well as employment in other high-risk settings (e.g., food processing plants) have, in some areas (e.g., Dallas, Texas; San Francisco, California), accounted for over 50% of positive COVID-19 cases (Fernandez & Weiler, 2020; Lucia, 2020).

For Black Americans, who are disproportionately represented in essential, frontline, and other higher risk occupations (Sim, 2020; Thompson, 2020), these data suggest potential increased risk for COVID-19 exposure and infection. Although studies have primarily centered on COVID-19 risk exposure among healthcare workers, research suggests a number of other frontline and essential occupations have been associated with greater risk exposure, including protective service, public transit workers, retail and service staff (Sim, 2020; Thompson, 2020). The disproportionate number of Black Americans employed as frontline and essential workers not only suggests increased risk of COVID-19 exposure and infection; but, also reflects the effects of existing social and racial inequities, which are being exacerbated during the COVID-19 pandemic (Dubay et al., 2020; Karaye, & Horney, 2020; Smith et al., 2020).

Black and Latino/a Americans are more likely than their Asian and White counterparts to work in jobs that are characterized by nontraditional shifts, longer work hours, and high demand/low control work conditions (Okechukwu et al., 2014; Williams, 2008). Following the job demands-resources model (Demerouti et al., 2001), increased job demands, without appropriate supports and resources, are linked to elevated stress and diminished well-being (National Sleep Foundation, 2010; Lee et al., 2019; Okechukwu et al., 2014). Amidst the COVID-19 pandemic, this heightened stress can increase the likelihood of mental health-related issues, such as PTSD, depression, and anxiety (Bao et al., 2020; Rajkumar, 2020; Williamson et al., 2020).

General and studies specific to Black American men have reported that stress and subsequent physiological responses can lead to disrupted sleep patterns (Matthews et al., 2013; Rodriguez et al., 2008; Williams, 2008). Studies examining work-related stress among essential and frontline workers, more broadly and during the COVID-19 pandemic have linked these conditions to greater sleep disturbance and poorer sleep quality (El-Hage et al., 2020; McKibbin & Fernando, 2020). Research has indicated that sleep problems can co-occur with other mental health-related disorders (Cain-Shields et al., 2020; Williams, 2008). Further, work conditions may contribute to overall health and life expectancy (Goh et al., 2015; Lee et al., 2019; Okechukwu et al., 2014). Goh and colleagues (2015) reported that workplace conditions contributed to annual mortality rates of Black men, suggesting the need to understand underlying and related factors (Goh et al., 2015).

Beyond impacts on psychological adjustment and sleep patterns, the day-to-day stressors associated with employment during an ongoing pandemic can *spillover* into familial life (Brock & Laifer, 2020; Spinelli et al., 2020). Work-family spillover frameworks posit that participation and experiences in one domain (i.e., work), can impact or spill over into another domain (i.e., family), affecting participation in, experiences, or outcomes in that domain (Pleck, 1995; Staines, 1980). Though much attention has been given to understanding these effects among parents who have been working from home or remotely, examining the potential impacts on parents that have continued to work outside the home during COVID-19 remains a critical line of inquiry. Reduced work flexibility, greater demand, and pace of the workday, which has characterized many essential and frontline employment settings, can influence family dynamics. Increased stress experienced by essential and frontline workers has been associated with greater levels of parenting stress and family discord (Broman et al., 2000; Minnotte et al., 2015). Previous research has indicated that there are race-related differences in work-family spillover and related conflict. Studies have indicated that Black and White men reported significantly more work-family conflict than Hispanic men and, after controlling for relevant variables (i.e., age, income, education, and work characteristics) (Ammons et al., 2017). Investigations have indicated that Black men report greater work-family spillover than Black women (Roehling et al., 2005). In the context of the COVID-19 pandemic, work-spillover effects may be exacerbated.

As this investigation turns attention to understanding how work contexts may shape individual well-being and family processes among Black men, one must acknowledge the legacy of earlier, deficit-focused and biased perspectives that have often mischaracterized Black fathers as unengaged in parenting and broader family processes. More contemporary perspectives (e.g., Coles et al., 2010; Johnson, 2001; McAdoo & McAdoo, 2002; Pate, 2010) on Black fatherhood have sought to situate parenting and involvement within broader social and ecological contexts. These perspectives have emphasized the need to understand transactional and interactional processes related to Black fatherhood and family formation. Lemmons and Johnson (2019) apply a critical race theoretical lens to highlight the multiplicative factors and experiences that shape the paternal involvement of Black men, including the intersection between race and social policy. Using this perspective as a framework, broader economic and work contexts shape, not only the individual well-being of Black men, but also have implications for their paternal involvement as well as the health and well-being of their families (Lemmons & Johnson, 2019). As we situate these broader processes within the COVID-19 pandemic, two demographic trends further support this study’s focus on Black fathers’ work contexts, individual well-being, and family dynamics. First, data indicate that Black and Latino/a Americans, in comparison to their White and Asian counterparts, have been more likely to continue working outside of the home during the COVID-19 pandemic (Gould & Shierholz, 2020). Additionally, studies indicate that Black Americans, including Black men, are generally overrepresented in occupations that are higher stress, lower control, and occupationally hazardous (Williams, 2008). These factors may increase exposure to COVID-19 as well as adversely impact individual well-being and family dynamics (McLafferty & Preston, 2019; Seabury et al., 2017; Williams, 2008).

Grounded in job demands-resources model (Demerouti et al., 2001) and applying a critical race lens (Lemmons & Johnson, 2019), this investigation examines how increased perceptions of occupational risk and demands are related to Black fathers’ well-being. First, as there is a continued need for descriptive data around COVID-19, in relation to potential contextual risk factors, this investigation examines how employment context is related to personal COVID-19 risk among our sample of Black fathers. Second, this study investigates the association between COVID-19 work context (e.g., working/not working in a higher-risk context for contracting COVID-19) and Black fathers’ psychological wellbeing (e.g., depressive and anxiety symptoms) and sleep health. Third, this study applies components of work-family spillover frameworks (Mennino et al., 2005) to better understand how work environments can spill over into familial contexts during the COVID-19 pandemic. In particular, this study investigates potential health implications for family members (e.g., likelihood of a COVID-19 diagnosis) and pandemic-related correlates of family stress. We hypothesize that higher-risk employment contexts during the COVID-19 pandemic will be associated with poorer health outcomes and psychological well-being among Black American men as well spillover into their family contexts.

## Method

### Participants

Data for this study come from a multiregional online panel sample of Black American fathers, which examined parenting and wellbeing within the context of COVID-19. Participants resided in all regions of the United States—(1) Southeast (39%; *n* = 182); (2) Northeast (22%; *n* = 102); (3) Midwest (17%; *n* = (77); and (4) West (21%; *n* = 105). Data collection occurred from June to July 2020, a time period of continuous increase in the number of COVID-19 cases across the United States. Participants were 498 fathers (*M* = 36.63; *SD* = 11.00) with children between the ages of 8 and 17 years of age (*M* = 12.36; *SD* = 2.85). Due to the study’s focus on work contexts, unemployed, and retired participants were excluded from analyses, resulting in a final sample of 466 fathers. Approximately 67% (*n* = 311) of the sample were currently partnered (married and living together; living together, not married) and 33% (*n* = 154) currently single. Twenty-nine percent of fathers had earned a high school education, equivalent or less (*n* = 129), 21% an associate/technical degree (*n* = 88), and 52% (*n* = 236) a college or advanced degree. Eighty-six percent (*n* = 394) of fathers indicated being currently employed and 14% (*n* = 68) temporarily laid off from work. Thirty-one percent (*n* = 137) of the sample reported working in contexts at high risk for contracting COVID-19. Fourteen percent (*n* = 64) of the sample did not have health insurance. Descriptive statistics and frequencies are presented in Table 1.

**Table 1.** Frequencies of Demographic Variables.

	Full sample	Higher risk work context	Lower risk work context
Father’s age			
Mean	39.32	38.99	39.49
*SD*	9.54	9.09	9.69
Father’s education			
High school diploma or less	129 (29%)	42 (32%)	82 (27%)
Associate/vocational/tech degree	88 (19%)	28 (21%)	58 (19%)
Bachelors or advanced degree	236 (52%)	63 (47%)	169 (55%)
Employment status			
Currently employed	394 (86%)	118 (87%)	267 (85%)
Temporary layoff	68 (14%)	18 (13%)	47 (15%)
Marital/partner status			
Currently partnered	311 (67%)	84 (62%)	220 (69%)
Not currently partnered	154 (33%)	52 (38%)	97 (31%)
Region of country			
Midwest	77 (16%)	24 (18%)	52 (16%)
Northeast	102 (22%)	36 (27%)	64 (20%)
Southeast	182 (39%)	48 (35%)	128 (41%)
West	105 (23%)	28 (21%)	74 (23%)
Health insurance status			
Uninsured	64 (14%)	22 (16%)	42 (13%)
Insured	401 (86%)	114 (84%)	275 (87%)
Personal Covid-19 diagnosis			
No	313 (68%)	78 (58%)	229 (72%)
Yes	147 (32%)	56 (42%)	88 (28%)
Family member COVID-19 diagnosis			
No	354 (79%)	87 (67%)	262 (84%)
Yes	96 (21%)	42 (33%)	50 (16%)
Number of children in household			
Mean	3.00	2.93	3.03
SD	1.37	1.22	1.43

*Note*. Due to missing data, frequency totals may vary across variables.

### Procedure

After obtaining human subjects approval through the University’s Internal Review Board (University of North Carolina at Chapel Hill, #191156), fathers were recruited via an online Qualtrics Panel study. Qualtrics Panels, which is a platform for recruitment, identifies eligible participants for study participation. Recent studies have highlighted that online panel studies provide robust data, comparable to other recruitment methods and increase participation of harder to recruit populations in research studies (Ibarra et al., 2018). Before completing the survey, participants signed a web-based consent form and verified eligibility criteria (African American/Black father). Surveys took approximately 25–30 min to complete. Questions included a range of demographic variables, contextual factors, parenting, and psychosocial factors. Additionally, measures were included to assess parenting and psychological well-being during the COVID-19 pandemic. Attention checks were included in surveys and quality checks were initially completed by Qualtrics Panels and again by the research team. Fathers were compensated for participation after study completion. Compensation was determined by Qualtrics Panels (not the principal investigator) based upon the length and difficulty of the survey. After completion, participants had the option of choosing among monetary compensation (less than $10), airline miles, or another comparable gift.

### Measures

#### COVID-19 Risk Work Context

One dichotomous question was used to assess fathers’ work context: (1) During the pandemic, are/were you working in a job that you considered to be high risk for contracting COVID-19 (0 = no; 1 = yes).

#### COVID-19 Health Indicators

Two items from the Coronavirus Impact Scale (Stoddard & Kaufman, 2020) assessed COVID-19 health impacts: (1) personal diagnosis (0 = no; 1 = yes) and (2) number of immediate family members diagnosed (e.g., spouse/partner, children, parents; 0 = no; 1 = yes).

#### Psychological Functioning

The short-form of the Patient-Reported Outcomes Measurement Information System (Pilkonis et al., 2011) (PROMIS) was used to measure (1) depressive symptoms (4 items; α = .92; “In the past 7 days, I felt hopeless.”; 1 = never; 5 = always) and (2) anxiety symptoms (4 items; α = .91; “In the past 7 days, I found it hard to focus on anything other than my anxiety.”; 1 = never; 5 = always). The validity of the short-form scale has been demonstrated across multiple samples (Pilkonis et al., 2011; Yu et al., 2012).

#### Sleep Health

Two indicators of sleep health (Pilkonis et al., 2011) were examined—1) sleep disturbance (2 items; α = .84; 1 = not at all; 5 = very much; “In the past 7 days, I had a problem with my sleep.”) and 2) sleep quality (1 item; “In the past 7 days, my sleep quality was very poor (1) to very good (5).”).

#### COVID-19 Family and Household Stress

One item from the COVID-19 Exposure and Family Impact Survey (CEFIS) (Center for Pediatric Traumatic Stress, 2020) measured perceived family impacts of the COVID pandemic (“In general, how has the COVID-19 pandemic affected parenting?”). Responses were on a 4-point Likert scale (1 = made it a lot better; 4 = made it a lot worse). Also, one item from the Coronavirus Impact Scale (Stoddard & Kaufman, 2020) measured fathers’ perceptions of family discord during the COVID-19 pandemic (“How much has the COVID-19 pandemic contributed to stress or discord in your family?”; 1 = none; 4 = severe).

## Data Analytic Strategy

Frequencies were used to provide prevalence information on fathers’ COVID-19 health outcomes (personal and family member diagnosis). T-tests examined mean differences between fathers’ work context and outcomes (e.g., family stress; psychological functioning; sleep health). Adjusting for demographic variables (age; health insurance status; region; work status; work hours; education level; partner status), binomial logistic (e.g., odds ratio of a personal and immediate family member COVID-19 diagnosis) and multiple regressions (e.g., family stress; psychological functioning; sleep health) were estimated to examine the association among fathers’ COVID-19 employment context, health- and family-related outcomes. Across demographic and core study variables, missing data ranged from 0% to 3.6%.

## Results

### Post-hoc Power Analysis

Given our sample size of 466, analyses revealed that the statistical power for the group comparisons was .68 for detecting a small effect and over .99 for detecting a moderate or large effect. The sample size (*n* = 466), 10 predictors and a *p* < .05 significance level were used as criteria for estimating a post-hoc power analysis for our regression analyses. For outlined regression analyses, the statistical power exceeded .99 for detecting a small (*f* ^2^ = .20), moderate (*f* ^2^ = .40), and large effect (*f* ^2^ = .80).

### High-Risk Work Contexts and Self-Reported COVID-19 Diagnoses

**Figure 1.**
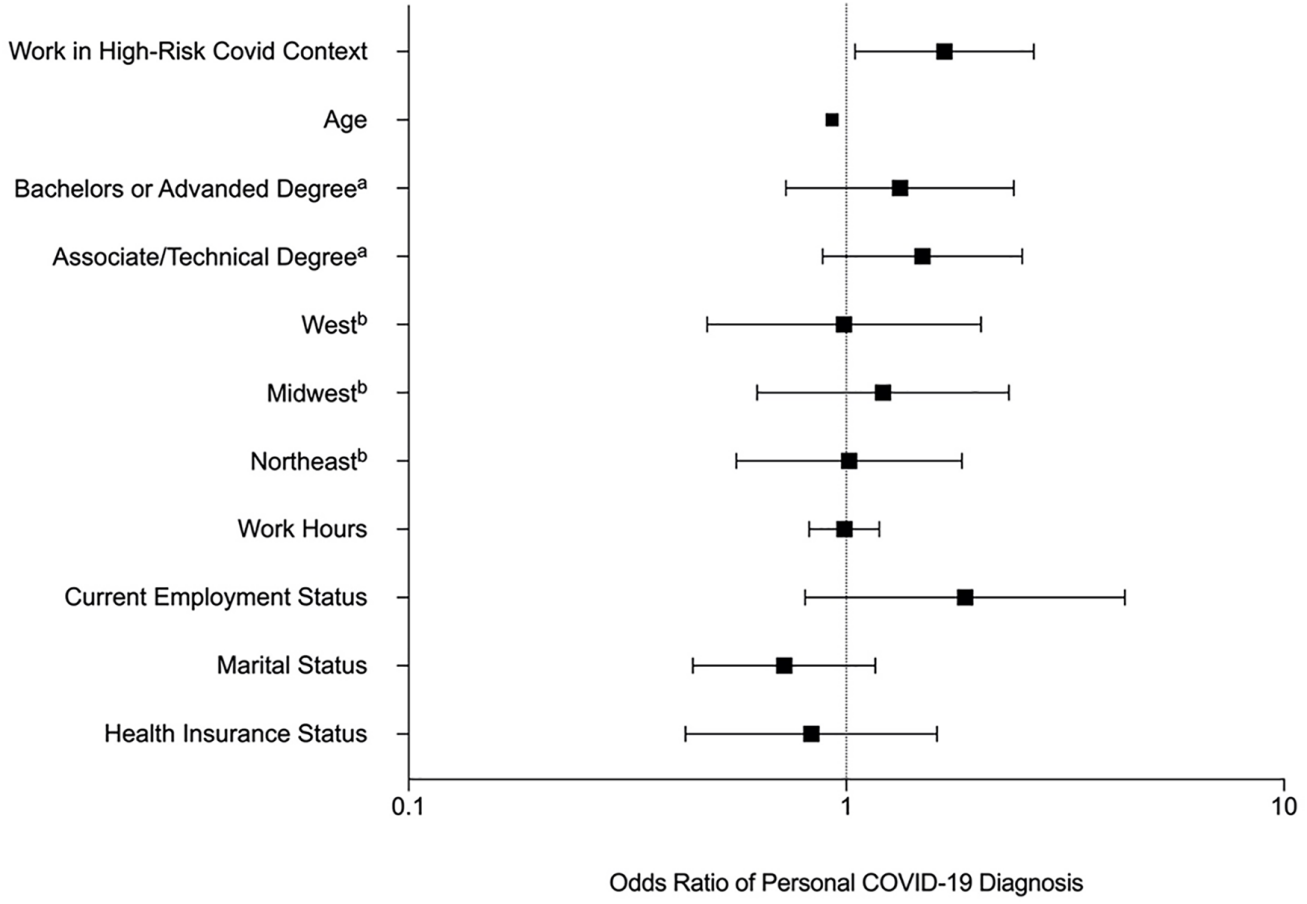
Forest Plot Based on Odds Ratio of COVID-19 Diagnoses. ^a^referent category = high school diploma or less. ^b^referent category = Southeast region of the United States.

**Figure 2.**
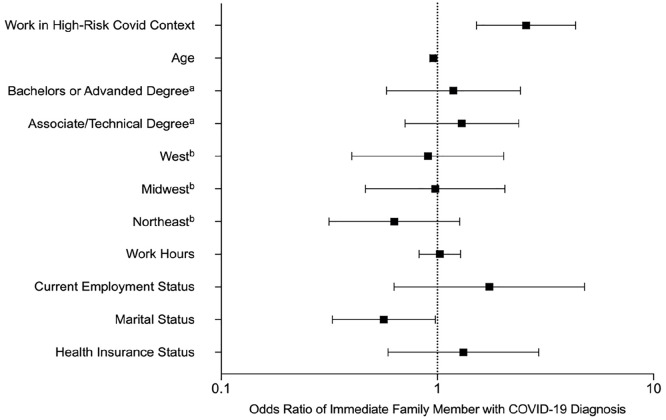
Forest Plot Based on Odds Ratio (Adjusted) of an Immediate Family Member with a COVID-19 Diagnosis ^a^referent category = high school diploma or less. ^b^referent category = Southeast region of the United States.

As presented in Table 1, 32% (*n* = 147) of fathers reported a personal diagnosis of COVID-19. Of those reporting a diagnosis, 35% (*n* = 51) reported mild (i.e., symptoms managed at home), 47% (*n* = 70) reported moderate (i.e., symptoms severe; brief hospitalization) and 18% (*n* = 26) reported severe symptoms. Approximately 21% (*n* = 90) of the sample indicated that at least one immediate family member had been diagnosed with COVID-19. A binary logistic regression was estimated to examine whether this difference held after controlling for key demographic variables (age; health insurance status; region; employment status; education level; partner status; work hours). Results indicated that the odds of a diagnosis for fathers working in a higher-risk context for contracting COVID-19 was 1.68 times that of fathers who reported not working in a high-risk context for contracting COVID-19 (95% CI: 1.05, 2.68). Additionally, the odds a family member contracting COVID-19 for fathers working in a higher-risk context for contracting COVID-19 was 2.58 times that of fathers who reported not working in a higher-risk context for contracting COVID-19 (95% CI: 1.52, 4.36). All binomial logistic regression estimates are presented in Table 2. Additionally, forest plots based on the adjusted odds ratio of a personal and immediate family COVID-19 diagnosis are shown in Figures 1 and 2.

**Table 2.** Adjusted Odds Ratio Estimating Personal and Family COVID-19 Diagnosis.

	Personal diagnosis		Family member diagnosis	
	Odds ratio	95% CI	Odds ratio	95% CI
Work in higher-risk COVID-19 context	1.68^a^	1.05–2.68	2.58^c^	1.52–4.36
Age	0.93^c^	0.90–0.95	0.95^b^	0.92–0.98
Education^‡^				
High school education or less	1		1	
Associate/technical degree	1.49	0.88–2.52	1.30	0.71–2.38
Bachelors or advanced degree	1.33	0.73–2.41	1.19	0.58–2.42
Region^§^				
Southeast	1		1	
Northeast	1.02	0.56–1.84	0.63	0.32–1.27
Midwest	1.21	0.63–2.35	0.98	0.47–2.05
West	0.99	0.48–2.03	0.90	0.40–2.03
Work hours	0.99	0.82–1.19	1.03	0.82–1.28
Current employment status	1.87	0.81–4.33	1.74	0.63–4.79
Marital status	0.72	0.45–1.17	0.57^a^	0.33–0.98
Health insurance status	0.83	0.43–1.61	1.32	0.59–2.94

*Note*. ^a^*p*< .05; ^b^*p*< .01; ^c^*p*<.001; ^‡^referent category = high school education or less; ^§^referent category = Southeast Region of United States.

### Higher-Risk COVID-19 Work Contexts and Psychological Functioning

Independent samples *t*-tests revealed that there were group differences in fathers’ reported depressive (*t*(452) = −3.865, *p* < .001) and anxiety symptoms (*t*(452) = −4.421, *p* < .001). Fathers working in higher-risk contexts for contracting COVID-19 reported greater depressive (*M =* 2.47; *SD =* 1.24, *p <* .001) and anxiety symptoms (*M =* 2.70; *SD =* 1.12, *p <* .001) than those not working in higher risk COVID-19 contexts (*M*_depressive_*=* 2.03; *SD =* 1.09; *M*_anxiety_*=* 2.22; *SD =* 1.07). A regression analyses, controlling for a number of key demographic variables (e.g., age; health insurance status; region; education level; work hours; partner status) indicated that fathers’ employment in higher-risk for contracting COVID-19 reported greater anxiety (B = .43; *SE* =.11, *p* < .001) and depressive symptoms (B = .41; *SE* =.11, *p* = .001). Multiple regression coefficients for all variables are presented in Table 3.

**Table 3.** Multiple Regression Estimates Predicting Fathers’ Wellbeing and Family Outcomes.

	Family discord	COVID-19 parenting	Depressive symptoms	Anxiety symptoms	Sleep disturbance	Sleep quality
COVID-19 work context	.40 (.10)^c^	.15 (.09)	.41 (.11)^c^	.43 (.11)^c^	.45 (.13)^c^	−.19 (.11)^‡^
Age	−.03 (.01)^c^	.01(.00)	−.04 (.01)^c^	−.04 (.01)^c^	−.03 (.01)^c^	.01 (.01)
Education	−.05 (.06)	−.04(.05)	−.05 (.06)	−.03 (.06)	.05 (.07)	−.03 (.06)
Northeast region^§^	−.17 (.12)	.11 (.10)	.16 (.14)	.27 (.13)^a^	−.04 (.15)	−.09 (.14)
Midwest region^§^	.00 (.14)	−01(.11)	.24 (.15)	.13 (.14)	.02 (.17)	.02 (.15)
West region^§^	.15 (.16)	−.12 (.13)	.07 (.14)	.05 (.13)	−.04 (.15)	.00 (.14)
Work hours	.05 (.04)	−.01 (.03)	.03 (.04)	.04 (.04)	.02 (.05)	.04 (.04)
Current employment status	−.32 (.16)^a^	−.07 (.13)	−.12 (.17)	.04 (.16)	−.19 (.19)	.19 (.17)
Partner status	.14 (.11)	−.03 (.09)	−.24 (.12)^a^	−.09 (.11)	−.04 (.13)	−.02 (.12)
Number of children in household	.05 (.03)	−.01 (.03)	.05 (.04)	.01 (.04)	−.04 (.04)	.11 (.04)^b^
Health insurance status	.02 (.14)	−.19 (.12) ^‡^	−.20 (.16)	−.28 (.15)^‡^	.09 (.17)	−.01 (.16)
*F*	5.71^c^	1.23	7.55^c^	8.49^c^	3.07^b^	1.80^‡^
*df*	10, 421	11, 424	10, 426	10, 426	10, 426	10, 426
*R* ^2^	.11	.01	.14	.16	.05	.02

*Note*. ^‡^*p* < .10; ^a^*p* < .05; ^b^*p* < .01; ^c^*p* <.001; ^§referent^ = Southeastern Region of the United States; COVID-19 work context (0 = lower risk work context for contracting COVID-19; 1 = higher risk context for contracting COVID-19); current employment status (0 = temporary lay-off; 1 = currently employed); partner status (0 = not currently partnered; 1 = currently partnered); health insurance status (0 = uninsured; 1 = insured)

### High-Risk COVID-19 Work Contexts and Sleep Health

There were group differences in sleep disturbance, *t*(452) = −3.643, *p* =.001); but, not overall sleep quality, *t*(452) = 1.543, *ns*). Fathers working in higher-risk COVID-19 contexts reported greater sleep problems, such that fathers in higher-risk contexts reported greater sleep disturbance (*M =* 2.86; *SD =* 1.28, *p <* .001) than those not working in higher-risk contexts (*M =* 2.46; *SD =* 1.16). Adjusted regression analyses (see Table 3 for all estimates) revealed that fathers’ employment in higher-risk for contracting COVID-19 was associated with greater sleep problems (B = .45; *SE* =.13, *p* < .001). However, fathers’ employment in higher-risk contexts for contracting COVID-19 was not related to overall sleep quality (B = −19; *SE* =.11, *ns*).

### High-Risk COVID-19 Work Contexts and Family Stress

Analyses revealed significant differences in family discord, *t*(447)= −3.864, *p* < .001; but, not COVID-19 parenting changes, *t*(450) = −1.673, *ns*. Fathers working in contexts at greater risk for contracting COVID reported greater family discord (*M =* 2.31; *SD =* 1.08) than fathers not working in higher risk contexts for contracting COVID-19 (*M =* 1.97; *SD =* .96). However, there were no significant group differences related to perceived COVID-19 related changes to parenting. Fathers working in higher-risk contexts (*M =* 2.11; *SD =* .86) did not report greater COVID-19 parenting changes than those not working in higher-risk contexts (*M =* 1.97; *SD =* .79). Multiple regression analyses (presented in Table 3) indicated that, after controlling for demographic variables (e.g., age; health insurance status; region; education level; work hours; partner status; number of children in household), working in a higher-risk context during the COVID-19 was associated with greater family discord (B = .40; *SE* =.10, *p* < .001). However, fathers’ work context was not related to COVID-19 parenting changes (B = .15; *SE* =.09, *ns*).

## Discussion

Given that Black men are over two times as likely to die from COVID-19 than White men and that they are overrepresented in higher risk, lower control occupations (Ayoubkhani et al., 2020; Sneed et al., 2020), this investigation turned attention to interrelations among Black American fathers’ work contexts, well-being, and family life during the COVID-19 pandemic. Specifically, the current study examined employment contexts’ COVID-19 risk in relation to their health and well-being. Additionally, this investigation explored whether COVID-19 work settings spillover into familial contexts. Several key findings emerged from this investigation. First, across the full sample, approximately 32% reported a personal diagnosis of COVID-19 and 21% reported an immediate family member (partner; children; parents) with a COVID-19 diagnosis. Unadjusted mean comparisons revealed that COVID-19 diagnosis likelihood differed between fathers working in higher versus those working in lower COVID-19 risk contexts. Further, after adjusting for several key demographic and contextual factors, we estimated that fathers working in a higher-risk context for contracting COVID-19 were almost 1.7 times the odds of reporting a personal diagnosis than those not working in higher risk contexts. Notably, among our multiregional sample of Black American fathers, only age and work context predicted likelihood of a COVID-19 diagnosis. In addition to age and work context, partner status predicted odds ratios of a personal family member reporting a diagnosis. Though research has not fully explained whether this risk is via direct exposure or the likelihood of immediate family members also working in higher risk occupations, such as essential and frontline workers, our study does suggest that COVID-19 work conditions may potential spillover into family domains.

Our results suggest that working in higher-risk contexts during a pandemic was associated with poorer wellbeing. In particular, fathers working in higher risk contexts reported greater depressive and anxiety symptoms, even after adjusting for other demographic and context variables. Work conditions have been linked to decreased psychological functioning and studies with Black American men have identified similar patterns. Although data are still emerging, COVID-19 work conditions, often characterized by lower wages, greater demands, longer work hours, and low control have been related to elevated stress. Higher levels of job stress, coupled with limited control of working conditions especially in high-risk jobs are associated with poor psychological health (Bao et al., 2020). Black American fathers who reported employment settings that were at a higher risk for contracting COVID-19, also reported greater sleep disturbance; but, not overall poorer sleep quality. For frontline and essential workers, who have experienced longer work hours and greater stress during the pandemic, sleep disturbance issues may be particularly relevant. Prior investigations reveal that both decreased psychological functioning and greater sleep problems are known gateways to poor and worsened overall physical and mental health among Black American men (Lee et al., 2019; Williams, 2008).

Fathers who were employed in jobs that were higher-risk for contracting COVID-19 reported greater family discord than fathers who did not work in higher-risk contexts. One potential pathway through which work contexts may impact family stress is through couple disagreements. Hostetler and colleagues (Hostetler et al., 2012) reported that couple disagreements partially mediated the relationship between negative work-family spillover and family satisfaction. Specifically, fathers who experience more negative-work family spillover engaged in more disagreements with their partners, which, in turn, led to poorer family satisfaction. This may be particularly heightened during a time where many essential and frontline workers must work extended hours and deal with extra restrictions on their jobs (i.e., use of personal protective equipment). This added work stress may spillover into their family lives and impact familial functioning. Future research should explore how the specific job conditions of essential and frontline workers during this pandemic may impact family routines and interactions. Findings from this study also indicated that there were no significant differences between these groups in how COVID-19 has potentially impacted their parenting. Due to school closures and the shift toward online learning, the ongoing COVID-19 pandemic has impacted parents across the United States. As such, the negative effects may not differ greatly between fathers who are or are not on the frontline or essential workers.

This study does have some limitations. First, this investigation was based upon self-report data. Although personal perspectives are critically important in assessing personal impacts, additional reporters (e.g., partner reports; supporting medical information) would have contributed to this study. This investigation’s cross-sectional design did not provide an opportunity to determine causality or examine longer-term effects. As the COVID-19 pandemic continues, studies are needed to assess the long-term impacts on frontline and essential workers. Additional information about fathers’ home and work contexts, including job or employment type and related characteristics would have provided a richer contextualization of essential and frontline occupations, work conditions and characteristics during the COVID-19 pandemic.

Despite the study limitations, our study has some important strengths. First, it provides critical descriptive data on COVID-19 prevalence rates. Our findings indicate that higher-risk employment contexts during COVID-19, such as essential and frontline occupations may be a health risk for Black American men. This health risk reflects existing health and employment inequities as well as how the COVID-19 pandemic context further exacerbates these inequities. Further, this investigation suggests that, while a focus on individual health outcomes is necessary, these effects can spillover into the familial domain. Given the biases and mischaracterizations of Black fathers and their familial involvement, this study helps to characterize how broader social and economic contexts shape both family processes and health-related outcomes.
